# Chromosome-Level Genome Assembly and Annotation of a Sciaenid Fish, *Argyrosomus japonicus*

**DOI:** 10.1093/gbe/evaa246

**Published:** 2021-02-23

**Authors:** Linlin Zhao, Shengyong Xu, Zhiqiang Han, Qi Liu, Wensi Ke, An Liu, Tianxiang Gao

**Affiliations:** 1 First Institute of Oceanography, Ministry of Natural Resources, Qingdao, Shandong, China; 2 Fishery College, Zhejiang Ocean University, Zhoushan, Zhejiang, China; 3 Wuhan Gooalgene Technology Co., Ltd., Wuhan, Hubei, China

**Keywords:** *Argyrosomus japonicus*, PacBio sequencing, de novo assembly, genome annotation, phylogenetic structure

## Abstract

*Argyrosomus japonicus* is an economically and ecologically important fish species in the family Sciaenidae with a wide distribution in the world’s oceans. Here, we report a high-quality, chromosome-level genome assembly of *A. japonicus* based on PacBio and Hi-C sequencing technology. A 673.7-Mb genome containing 282 contigs with an N50 length of 18.4 Mb was obtained based on PacBio long reads. These contigs were further ordered and clustered into 24 chromosome groups based on Hi-C data. In addition, a total of 217.2 Mb (32.24% of the assembled genome) of sequences were identified as repeat elements, and 23,730 protein-coding genes were predicted based on multiple approaches. More than 97% of BUSCO genes were identified in the *A. japonicus* genome. The high-quality genome assembled in this work not only provides a valuable genomic resource for future population genetics, conservation biology and selective breeding studies of *A. japonicus* but also lays a solid foundation for the study of Sciaenidae evolution.

**Significance**

Limited genetic and genomic information for marine species restricts breeding development and resources conservation. In this study, we obtained a chromosome-level genome assembly of *Argyrosomus japonicus*, which will contribute to research on the genomics, evolution, and conservation biology of this species.

## Introduction

The family Sciaenidae is one of the largest groups in the order Perciformes, comprising 66 genera with 291 species worldwide and 14 genera with 37 species in China (Zhu et al. 1963; Nelson 2006). *Argyrosomus japonicus* is a large-bodied fish in Sciaenidae that is widely distributed in estuaries and nearshore coastal waters (<100 m depth) of the Pacific and Indian Oceans surrounding Australia, South Africa, India, Pakistan, China, Korea, and Japan (Nakabo 2013). *Argyrosomus japonicus* was usually identified as *Nibea japonica* in previous studies in China, but this has been proven to be an invalid species name (synonym of *A. japonicus*) (Shen and Wu 1993; Xu 2010). The biology of *A. japonicus* has been well studied in Australia and South Africa, but little information is available from other areas of its distributional range (Lo et al. 2015). Due to the long-distance geographical isolation and different life histories of its populations, the population structure of *A. japonicus* might differ among regions.

In recent decades, overfishing and habitat degradation have led to a dramatic decrease in the population resources of *A. japonicus* in the wild ocean (Silberschneider et al. 2009). To cope with the declining wild stocks and growing seafood demand, the aquaculture of *A. japonicus* has been initiated in various areas worldwide (Wei et al. 2012). Due to its characteristics of disease resistance and rapid growth, this species is regarded as an important mariculture species in China, South Africa, and Australia (Bolton et al. 2013). Increased productivity achieved via genetic improvement with selective breeding has been a key factor facilitating the development of major aquaculture industries. To date, genomes from six species of sciaenid fishes have been sequenced including *Larimichthys crocea*, *Larimichthys polyactis*, *Collichthys lucidus*, *Miichthys miiuy*, *Sciaenops ocellatus*, and *Nibea albiflora* (detailed information in supplementary table S1, Supplementary Material online). However, the limited genetic information of *A. japonicus* restricts its further breeding and germplasm conservation. Therefore, there is an urgent need to obtain high-quality chromosome-level genome resources for *A. japonicus* to facilitate its selective breeding and reveal its phylogenetic relationships within its distribution region.

In the present study, we constructed a chromosome-level genome assembly of *A. japonicus* by combining Illumina short reads, PacBio long reads, and Hi-C sequencing data. We expected that this chromosome-level genome will promote studies on the selective breeding and population genetics of *A. japonicas*.

## Materials and Methods

### Sample Collection and Sequencing

A male individual of *A. japonicus* was sampled from a breeding farm in Zhoushan City, Zhejiang Province, for genome sequencing. Fresh muscle, eye, skin, gonad, gut, kidney, liver, brain, and blood samples were collected and quickly frozen in liquid nitrogen for 1 h before storage at –80 °C. Muscle tissue was used for DNA sequencing, whereas all tissues were used for transcriptome sequencing.

Total genomic DNA was extracted from fresh muscle using the standard phenol/chloroform method and sequenced using the PacBio Sequel II platform (for genome assembly) and the Illumina NovaSeq platform (for genome surveying and base correction after assembly). To obtain a chromosome-level genome, a 0.2-ml blood sample from the same individual was used for Hi-C library construction and sequencing with the same method used in a previous study (Gong et al. 2018). RNA was extracted from different tissues using the TRIzol Reagent (Invitrogen), then mixed in equal amounts and subjected to RNA-seq using the Illumina NovaSeq platform.

### Genome Assembly and Assessment

Before genome assembly, the size, heterozygosity, and repeat content of the *A. japonicus* genome were estimated through k-mer analysis with jellyfish (Marcais and Kingsford 2011).

To assemble the genome of *A. japonicus*, we applied Canu to analyze PacBio long reads (Koren et al. 2017). To correct random sequencing errors in the assembled genome, two steps of genome sequence polishing were applied: We first used the Quiver algorithm to polish the genome using PacBio long reads (Chin et al. 2013), and another round of genome-wide base-level correction was performed using Pilon with the Illumina clean reads (Walker et al. 2014). For chromosome-level scaffolding, the cleaned Hi-C reads were mapped to the assembled genome using BWA, and only uniquely mapped read pairs were considered for subsequent analysis (Li and Durbin 2009). We then used LACHESIS to cluster, order, and orient the assembled contigs (Burton et al. 2013). To evaluate the quality of the assembled genome, its completeness and accuracy were assessed via sequenced read mapping and benchmarking universal single-copy ortholog (BUSCO) analysis (Seppey et al. 2019).

### The Annotation of Repetitive Elements

Repeat sequences were identified in the *A. japonicus* genome via a combination of homology-based and de novo approaches. First, we used Tandem Repeats Finder (TRF) to detect tandem repeats and RepeatModeler to detect repeat sequences in the assembled genome (Benson 1999; Tarailo and Chen 2009). Based on the Repbase library, we used RepeatMasker and RepeatProteinMasker to annotate repeat elements and TE proteins, respectively (Bao et al. 2015).

### Gene Prediction and Functional Annotation

For gene structure prediction, we used a combination of de novo, homology-based and transcriptome-based strategies to predict genes in the *A. japonicus* genome. The de novo approach was implemented using Augustus (Stanke et al. 2008). For homology-based prediction, TBlastN was used to align protein sequences from big head croker (*C. lucidus* from Sciaenidae), larger yellow croaker (*L. crocea* from Sciaenidae), ocellaris Clownfish (*Amphiprion ocellaris* from Pomacentridae), spiny chromis (*Acanthochromis polyacanthus* from Pomacentridae), eastern happy (*Astatotilapia calliptera* from Cichlidae), and Climbing perch (*Anabas testudineus* from Anabantidae) to the assembled genome of *A. japonicu*s (Gertz et al. 2006). Then, the transcriptome sequence reads were aligned to the genome using the TopHat package, and gene structure was predicted using Cufflinks (Trapnell et al. 2009; Ghosh and Chan 2016). Finally, all gene models were merged, and redundancy was removed wit MAKER (Cantarel et al. 2007). 

### Phylogenetic Analysis of *A. japonicus*

To reveal the phylogenetic relationships with other species, we downloaded the protein sequences of *C. lucidus* (GCA_004119915.1), *L. crocea* (GCF_000972845.2), *Takifugu rubripes* (GCF_901000725.2), *Oryzias latipes* (GCF_002234675.1), *Lates calcarifer* (GCF_001640805.1), *Gasterosteus aculeatus* (GCA_006229165.1), *Dicentrarchus labrax* (GCA_000689215.1), and *Danio rerio* (GCF_000002035.6) from the NCBI database, and *Sillago sinica* (http://doi.org/10.5524/100490) from GigaScience database. The proteome sets of all species were analyzed with OrthoMCL to construct different types of orthologues (Li 2003). These single-copy orthologues were aligned using MUSCLE, and phylogenetic trees were generated with RAxML with 500 bootstrap replicates (Edgar 2004; Stamatakis 2014). MCMCTREE was applied to obtain estimates according to the divergence time-based approximate likelihood calculation method using molecular clock data from the TimeTree database (http://www.timetree.org/), including data for *L. crocea* and *D. labrax* (87–105 Ma), *L. crocea* and *L. calcarifer* (94–115 Ma), *L. crocea* and *O. latipes* (104–145 Ma) (Yang 1997; Hedges et al. 2006). 

## Results and Discussion

### Genome Assembly and Assessment

The details of the sequencing data obtained in the present study are listed in supplementary table S2, Supplementary Material online. The 17-mer frequency of short reads followed a Poisson distribution, with the highest peak occurring at a depth of 93 (supplementary fig. S1, Supplementary Material online). The estimated genome size was ∼675 Mb, the heterozygosity rate of the genome was 0.21%, and the repeat content of the genome was 35.47%.

Using Canu, we obtained an *A. japonicus* genome of 791 Mb with 1,984 contigs and a contig N50 of 13.1 Mb. After correcting the random sequencing errors in the assembled genome, the genome assembly of *A. japonicus* contained 674 Mb of sequences within 282 polished contigs, with a contig N50 of 18.4 Mb, and the overall GC content was 41.20%. Furthermore, 271 assembled contigs (98.8% of genome) were successfully clustered into 24 chromosome groups. Finally, we obtained a high-quality chromosomal-level genome with a total size of 674 Mb (supplementary table S3, Supplementary Material online), and the contig N50 and scaffold N50 lengths were 18.4 and 29.4 Mb, respectively, which were longer than most of other fish in Sciaenidae. Furthermore, 98.22% of short reads were mapped to the assembled genome, which covered 99.95% of the assembly. Then, the PacBio long reads were mapped with BLASR, and 95.57% of long reads covered 99.86% of the assembled genome. Finally, BUSCO was also used to evaluate the completeness of the genome assembly; 97.77% of the “complete BUSCOs” were successfully identified in the assembly, and the proportion of “missing BUSCOs” was only 1.4%. These results demonstrate the high reliability and completeness of the reported genome assembly.

### Genome Repetitive Elements and Gene Prediction

After removing redundancies, a total of 217.2 Mb of sequences (32.24% of the *A. japonicus* genome) were identified as repeat elements. Among these repeat elements, DNA transposons were the main type, accounting for 16.24% (100.43 Mb) of the repeat elements. A total of 23,730 protein-coding genes were predicted in the present study (table 1). Among all protein-coding genes, 22,938 protein-coding genes, corresponding to 97.34% of the total predicted genes in the *A. japonicus* genome, were functionally annotated in at least one public database (supplementary table S4, Supplementary Material online).

**Table 1:** Gene Structure Prediction of the *Argyrosomus japonicus* Genome

Gene Set		Number	Average Gene Length (bp)	Average CDS Length (bp)	Average Exons Per Gene	Average Exon Length (bp)	Average Intron Length (bp)
De novo prediction	AUGUSTUS	28,447	11,628.52	1,434.27	8.16	175.77	1,423.80
Homologue prediction	*Collichthys lucidus*	63,173	7,412.39	883.72	4.09	216.31	2,115.92
	*Larimichthys crocea*	51,693	7,396.15	1,077.62	5.27	204.66	1,481.33
	*Amphiprion ocellaris*	48,436	7,640.05	1,056.38	5.36	196.95	1,508.73
	*Acanthochromis polyacanthus*	50,682	7,140.94	993.37	5.12	194.15	1,493.39
	*Astatotilapia calliptera*	50,080	8,179.76	1,107.74	5.28	209.8	1,652.34
	*Anabas testudineus*	50,589	8,103.9	1,063.8	5.32	199.79	1,627.96
RNA-seq	Cufflinks	14,679	16,664.61	1,895.99	11.91	310.89	1,188.27
Final	MAKER	23,730	15,378.24	1,664.78	9.81	269.66	1,445.04

### Phylogenetic Relationships of *A. japonicus*

**Fig. 1:**
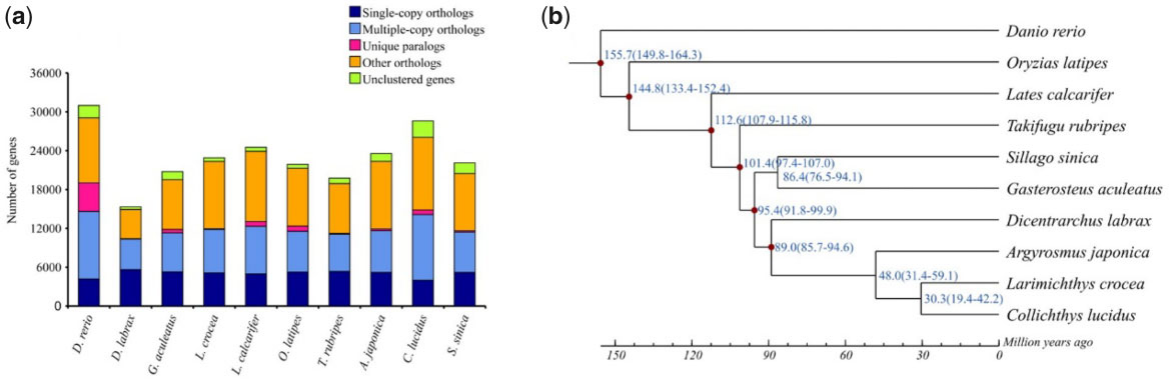
(*a*) Distribution of different types of orthologues in the selected representative. (*b*) Time tree and estimated divergence times of representative species based on single-copy orthologues.

Using OrthoMCL, we identified a set of 2,502 single-copy orthologues (fig. 1*a*). Then, the data matrix was applied to construct a phylogenetic tree (supplementary fig. S2, Supplementary Material online) and estimate the divergence time (fig. 1*b*). *Argyrosomus japonicus* diverged from the common ancestor with *C. lucidus* and *L. crocea* ∼48 Ma, and the divergence time between Sciaenidae and other teleosts (89.0 Ma) was similar to that reported in a previous study (∼90 Ma; Betancur et al. 2017).

## Conclusions

In this work, we applied a combined strategy involving Illumina, PacBio and Hi-C technologies for the de novo assembly of a chromosome-level genome for *A. japonicus*. We assembled the genome sequences into 282 contigs with a total length of 673.7 Mb and a contig N50 length of 18.4 Mb. By using Hi-C data, the contigs were further ordered and clustered into 24 chromosomes with a total length of 665.4 Mb. This well-annotated chromosome-level whole-genome sequence should be a valuable resource for studies on the genomics, evolution, and conservation biology of *A. japonicus*.

## Supplementary Material

Supplementary data are available at *Genome Biology and Evolution* online.

## Supplementary Material

evaa246_Supplementary_Data
