# Correction: Chromosome-Level Genome Assembly and Annotation of a Sciaenid Fish, Argyrosomus japonicus

**DOI:** 10.1093/gbe/evac150

**Published:** 2022-10-26

This is a Correction to: Chromosome-Level Genome Assembly and Annotation of a Sciaenid Fish, *Argyrosomus japonicus, Genome Biology and Evolution*, Volume 13, Issue 2, February 2021, evaa246, https://doi.org/10.1093/gbe/evaa246

The Chromosome assemblies and annotations were not published with the original manuscript; they can now be found at https://doi.org/10.6084/m9.figshare.20486925.v1

These details have been corrected only in this correction notice to preserve the published version of record.

