# Supplementary material for: Chromosome-Level Genome Assembly and Annotation of a Sciaenid Fish, Argyrosomus japonicus
Source: Genome Biol Evol. 2021 Feb 23;13(2):evaa246. doi: 10.1093/gbe/evaa246 (PMC7874996; doi:10.1093/gbe/evaa246)
Supplement: evaa246_Supplementary_Data [file evaa246_supplementary_data.docx]

Table S1. Information of genome assemblies in Sciaenidae

| Scientific names | Assembly level | Genome size (Mb) | Scaffold N50  (bp) | Contig N50  (bp) | GeneBank ID |
| --- | --- | --- | --- | --- | --- |
| *Larimichthys crocea* | Chromosome | 657.94 | 27,037,660 | 277,487 | JRPU00000000 |
| *L. polyactis* | Scaffold | 694.45 | 146,739 | 8,079 | WMHY00000000 |
| *Collichthys lucidus* | Chromosome | 877.61 | 35,919,243 | 1,098,566 | SCMI00000000 |
| [*Miichthys miiuy*](https://www.ncbi.nlm.nih.gov/Taxonomy/Browser/wwwtax.cgi?mode=Info&id=240162&lvl=3&lin=f&keep=1&srchmode=1&unlock) | Scaffold | 619.30 | 1,145,539 | 81,271 | JXSJ00000000 |
| [*Sciaenops ocellatus*](https://www.ncbi.nlm.nih.gov/Taxonomy/Browser/wwwtax.cgi?mode=Info&id=76340&lvl=3&lin=f&keep=1&srchmode=1&unlock) | Chromosome | 684.63 | 25,657,701 | 151,930 | WIRC00000000 |
| *Nibea albiflora* | Scaffold | 574.47 | 2,154,052 | 55,145 | OOII00000000 |
| *Argyrosomus japonicus* | Chromosome | 673.73 | 29,444,495 | 18,456,125 | JADEYK000000000 |

Table S2. Summary of sequencing data used for *Argyrosomus japonicus* genome assembly.

| Library type | Sequencing platform | Read length (bp) | Clean data (Gb) | Sequence coverage (×) | Application |
| --- | --- | --- | --- | --- | --- |
| Short reads | Illumina Novaseq | 2×150 | 85.6 | 128 | Genome survey and base correction |
| Long reads | PacBio SEQUEL | 16,204* | 146.3 | 221 | Genome assembly |
| Hi-C | Illumina Novaseq | 2×150 | 63.9 | 95 | Chromosome construction |
| Transcriptome | Illumina Novaseq | 2×150 | 10.6 | --- | Genome annotation |

* average read length

Table S3. Summary of assembled 24 chromosomes of *A. japonicas*.

| Chromosomes | Length (bp) | Contig number | Gene number |
| --- | --- | --- | --- |
| chr1 | 35,187,532 | 9 | 983 |
| chr2 | 33,263,957 | 11 | 1,269 |
| chr3 | 32,138,189 | 16 | 1,140 |
| chr4 | 31,259,840 | 3 | 1,021 |
| chr5 | 30,886,943 | 20 | 1,023 |
| chr6 | 30,873,492 | 9 | 1,139 |
| chr7 | 30,733,076 | 3 | 907 |
| chr8 | 30,223,700 | 8 | 913 |
| chr9 | 30,198,048 | 9 | 1,007 |
| chr10 | 30,083,800 | 9 | 1,165 |
| chr11 | 29,444,495 | 6 | 1,159 |
| chr12 | 29,029,172 | 3 | 1,133 |
| chr13 | 28,374,698 | 10 | 1,100 |
| chr14 | 27,338,617 | 16 | 896 |
| chr15 | 26,996,654 | 8 | 998 |
| chr16 | 26,473,408 | 7 | 1,183 |
| chr17 | 25,823,706 | 15 | 870 |
| chr18 | 25,086,375 | 2 | 899 |
| chr19 | 24,851,000 | 2 | 888 |
| chr20 | 24,637,600 | 7 | 709 |
| chr21 | 23,953,200 | 3 | 935 |
| chr22 | 21,946,400 | 4 | 634 |
| chr23 | 21,578,200 | 3 | 820 |
| chr24 | 14,996,600 | 7 | 487 |

Table S4. Summary of functional annotation of protein-coding genes in the genome of *A. japonicas.*

| Type | Database | No. of annotated genes | Percent (%) |
| --- | --- | --- | --- |
| Total |  | 23566 |  |
| Annotated |  | 22938 | 97.34 |
|  | InterPro | 20908 | 88.72 |
|  | GO | 15862 | 67.31 |
|  | KEGG | 22643 | 96.08 |
|  | Swissprot | 19933 | 84.58 |
|  | TrEMBL | 22764 | 96.6 |
|  | Pfam | 20178 | 85.62 |
|  | NR | 22884 | 97.11 |
| Unannotated |  | 628 | 2.66 |


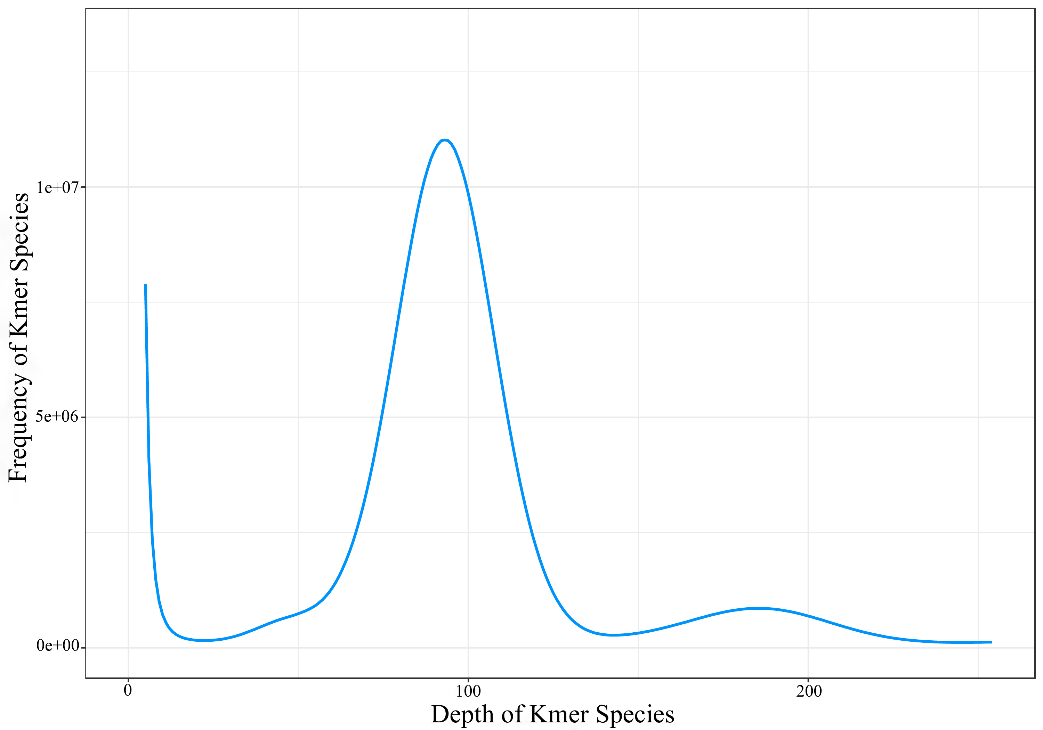


Figure S1. The 17-kmer count distribution for the genome size estimation.


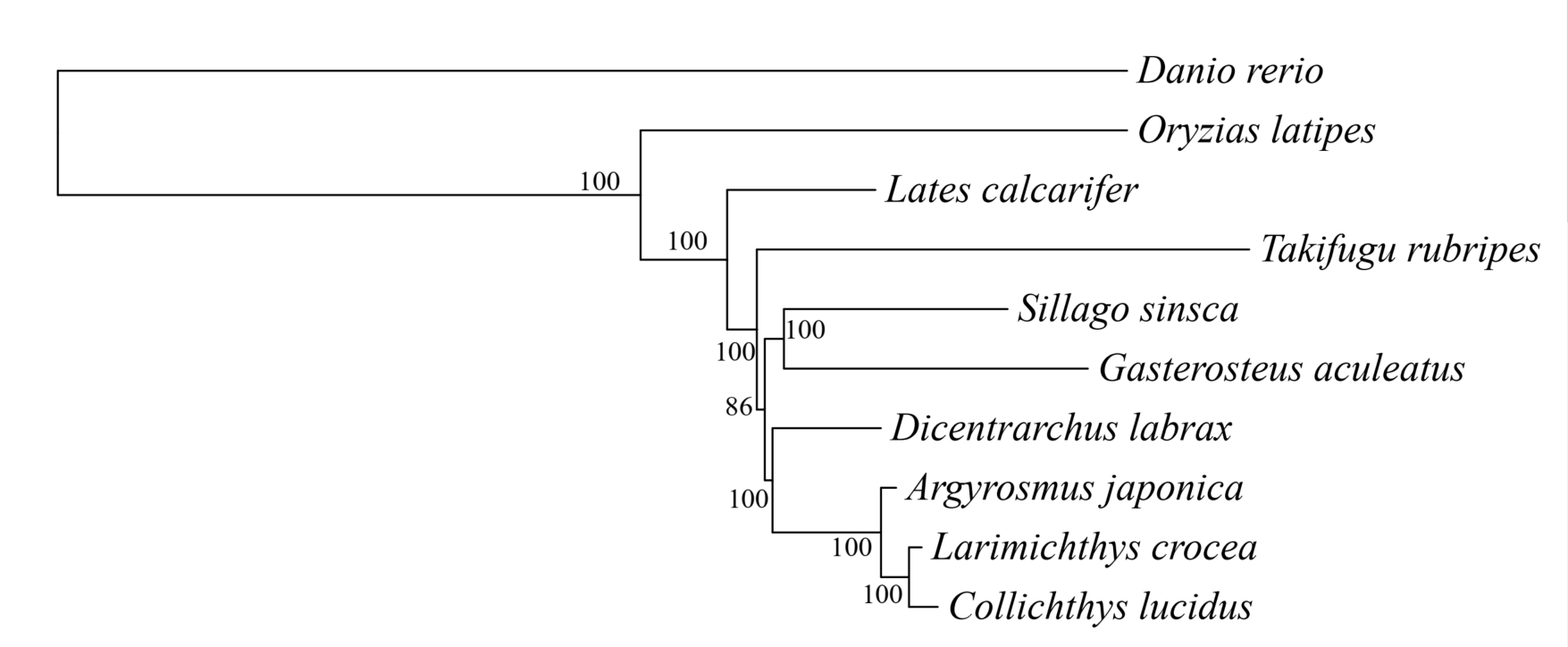


Figure S2. Phylogenetic tree of representative species based on single-copy orthologues. The bootstrap support values for the topology were on each node.
